# Influence of Chronic Periodontitis on the Long-Term Mortality and Cardiovascular Events in Kidney Transplant Recipients

**DOI:** 10.3390/jcm9061968

**Published:** 2020-06-23

**Authors:** Marta Wynimko, Magdalena Walicka, Yaroslav Sanchak, Dariusz Gozdowski, Anna Błach, Andrzej Więcek, Andrzej Śliwczyński, Edward Franek, Aureliusz Kolonko

**Affiliations:** 1 Department of Internal Diseases, Endocrinology and Diabetology, Central Clinical Hospital MSWiA, Wołoska 137, 02-507 Warsaw, Poland; mhwynimko@gmail.com (M.W.); m_walicka@wp.pl (M.W.); sanchaky@gmail.com (Y.S.); Edward.franek@cskmswia.pl (E.F.); 2 Department of Biometry, Warsaw University of Life Sciences, Nowoursynowska 166, 02-787 Warsaw, Poland; dariusz.gozdowski@sggw.pl; 3 Department of Cardiology, Medical University of Silesia, Francuska 20, 40-027 Katowice, Poland; anjablach@gmail.com; 4 Voxel SA Nuclear Medicine Center, Ceglana 35, 40-514 Katowice, Poland; 5 Department of Nephrology, Transplantation and Internal Medicine, Medical University of Silesia, Francuska 20, 40-027 Katowice, Poland; awiecek@sum.edu.pl; 6 Sattelite Campus, University of Humanities and Economics in Lodz, Wolność 2a, 01-018 Warsaw, Poland; andrzej.sliwczynski@nfz.gov.pl; 7 National Health Fund, Grójecka 186, 02-390 Warsaw, Poland; 8 Mossakowski Clinical Research Centre, Polish Academy of Sciences, Pawińskiego 5, 02-106 Warsaw, Poland

**Keywords:** kidney transplantation, periodontal disease, survival, mortality, death, cardiovascular complications, outcome, graft loss

## Abstract

Chronic periodontitis (CP) is associated with cardiovascular disease and mortality in different populations. The aim of this study was to examine an association of CP with hard endpoints in patients after kidney transplantation during a 15-year follow-up period. Study group consist of 117 patients (77M/40F, median age 44 years) divided into two subgroups: those with initially advanced CP (CPITN 3–4) and those with no or moderate CP (CPITN 0–2). All cardiovascular events, graft losses, and re-transplantations were recorded. All deaths were noted and verified, including those occurred after the return to dialysis therapy, the causes of death were identified. Cox regression with Firth’s penalized maximum likelihood models were used for data analysis. During the observation period, 49 deaths occurred. Advanced CP (*n* = 35) was not associated with overall mortality but was associated with increased risk of death with functioning graft (DWFG) [HR 3.54 (1.20–10.45); *p* < 0.05]. Risk of graft loss was not associated with CP status. In conclusion, an advanced CP was independently associated with increased risk of DWFG, but not all-cause or cardiovascular mortality after renal transplantation.

## 1. Introduction

Chronic periodontitis (CP) is an inflammatory disease of the oral cavity caused by the accumulation of bacterial plaque on the tooth surface. It manifests itself as pain, bleeding, and weakening of tooth-supporting structures with the eventual loosening and loss of teeth. CP causes local dental problems but also has systemic consequences [1]. Among others, it constitutes one of the factors that contribute to the development of chronic kidney disease (CKD), however, the relationship between those two entities seems to be bidirectional [2,3].

Patients suffering from CKD have significantly higher cardiovascular and all-cause mortality as compared to general population. It is also known that cardiovascular complications are the main cause of death in this population [4]. On the other hand, there is consistent and strong epidemiologic evidence that periodontitis also increases the risk of cardiovascular complications [5,6]. There are several possible mechanisms through which periodontitis and periodontal bacteria may affect multiple organs, including systemic bacteremia, cytokine release, and inflammation [1,7]. Bacterial pathogens, antigens, endotoxins, and inflammatory cytokines may contribute to the process of atherogenesis and thromboembolic events.

In kidney transplant recipients, some inflammatory markers were shown to be associated with death with a functioning graft [8,9]. Also, it was previously demonstrated by our group that severe CP is associated with an increased serum C-reactive protein (CRP) and the risk of patients’ death after kidney transplantation [10]. Moreover, the recently published systematic review indicates that a worse periodontal status may be associated with the largest ventricular mass, greater carotid thickness, lower graft survival, and higher mortality rate among kidney transplant recipients [11]. However, until now, only very few studies assessed the effect of periodontitis on patient or graft survival after kidney transplantation. Hence, the aim of this study was to evaluate the long-term influence of chronic periodontitis on cardiovascular events and overall mortality in kidney transplant recipients.

## 2. Materials and Methods

Originally, we investigated 199 unselected kidney transplant recipients, who were at least 12 months after kidney transplantation and still attended our out-patient clinic during the study enrollment period (2002–2003). At the start, any potential sources of infection were carefully screened through physical examination, blood tests, as well as radiologic and ultrasound imaging [10]. After that procedure, 82 patients were excluded, as they had been confirmed with other source of infection, such as chronic tonsillitis, sinusitis, kidney stones, cholelithiasis, or chronic gynecological infections. The final study group consisted of 117 (77 male) patients. The study protocol was prepared in accordance with the Declaration of Helsinki and was approved by the local Bioethics Committee. All study participants gave their informed consent.

In all study subjects, a dental examination was performed by one experienced dentist and the periodontal status according to the Community Periodontal Index of Treatment Needs (CPITN) [12] was assessed. The highest scoring CPITN sextant was recorded, and the subjects were divided into two subgroups: those with advanced CP (CPTIN 3–4) and those with no or moderate CP (CPTIN 0–2). The median time since transplantation to dental examination was 41 (28–76) months. In 2010, the second dental examination was performed in all subjects with a functioning graft.

Before transplantation, all patients were routinely screened and must have been dentally treated, if appropriate, according to the transplant center requirements. However, no detailed data are available concerning their dental status prior to kidney transplantation. After the transplant, no regular screening was provided, and each patient attended the dentist (or not) individually. Until the time of the study, no patient was treated due to periodontal disease.

In the present follow-up study, in order to assess the long-term influence of initial CP status on the clinical outcomes of both patients and kidney grafts, we collected information concerning the major study endpoints, effective on 31 October 2019. All patient deaths were noted and verified using the National Death Registry. Notably, we also recorded all deaths which occurred with a functioning graft and during the dialysis treatment. Furthermore, based on the original medical records, we clarified the particular cause of death, including cardiovascular or cerebrovascular episodes, infectious complications, malignancy, and accidental death. Additionally, we recorded all cardiovascular complications and procedures, including stent placement or coronary aortic bypass grafting (CABG), myocardial infarction, stroke (further major adverse cardiovascular events, MACE), based on the claims issued to the national health care cost payer.

Information about return to dialysis therapy and re-transplantations were also collected. After the loss of adequate kidney graft function, the general policy was to maintain the graft with diuresis, ceasing the calcineurin inhibitors and antimetabolite drug, and maintaining prednisone at a dose of 5–7.5 mg/day. The subsequent graftectomy was performed, if there were medical indications, i.e., clinical signs of rejection, recurrent urinary infections, or the clinical reason to stop steroids (due to cardiovascular disease, osteoporosis, etc.).

In patients with a functioning graft, we noted the current serum creatinine concentration and the presence of proteinuria.

### Statistical Analysis

The statistical analysis was performed using the Statistica 13.3 for Windows software package (Tibco Inc., Palo Alto, CA, USA), MedCalc v19.2.1 (MedCalc Software, Mariakerke, Belgium) and R software with the ‘coxphf’ package (https://cran.r-project.org/web/packages/coxphf/coxphf.pdf). Values are presented as means and 95% confidence intervals or frequencies. Variables with skewed distribution were presented as medians and Q25–Q75 values. The initial comparison of two groups based on the baseline CP status was performed using the Student’s *t*-test or χ^2^ test (for variables with normal distribution) or Mann–Whitney U test (for variables with not normal distribution). The univariate analyses were performed using linear logistic regression.

To evaluate the effect of CP on patient and kidney graft outcomes, survival analyses were performed using the Cox regression with Firth’s penalized maximum likelihood method. Models were built using the results of univariate analyses. The covariates included in the particular analyses are listed in the descriptions of figures. The significance was determined using the χ^2^ test. *p* values below 0.05 were considered as statistically significant.

Survival time is presented in years, where ‘0’ denotes the time of patient’s clinical examination when entering the study.

## 3. Results

### 3.1. Study Group

The baseline clinical characteristics of the study group is given in Table 1. Shortly, it consisted of 117 patients (77M/40F, median age 44 years), 35 of whom suffered from advanced CP. Patients in this subgroup were older and presented higher blood pressure. They were also characterized by greater left ventricular mass index (LVMI) and higher CRP levels. Both subgroups did not differ significantly in terms of kidney graft function, time since kidney transplantation, and immunosuppressive regimen.

During 15 years of follow-up, 49 patients died (41.9%) and 63 (53.8%) lost their kidney graft and returned to hemodialysis therapy. The causes of graft loss are given in Table 1. From those who lost their graft, 20 (31.7%) were then re-transplanted. Also, in 27 (42.9%) patients, graftectomy was performed. The detailed structure of deaths was analyzed. In 20 patients (40.8%), death was qualified as caused by underlying cardio- or cerebrovascular disease (CVD). There were also 12 (24.5%) infectious deaths, 10 (20.4%) deaths related to malignancy, and 5 (10.2%) accidental deaths. Furthermore, 1 patient died due to the advanced liver cirrhosis and in 1 patient the specific cause of death cannot be confirmed. Two patients died after being re-transplanted.

After 15 years of follow-up, in 37 patients with still functioning kidney graft, median serum creatinine concentration was 1.5 (interquartile range, 1.1–2.2) mg/dL. In a whole study cohort, including patients who have been re-transplanted, the current median serum creatinine concentration was 1.4 (1.1–2.0) mg/dL and only 3 patients had daily proteinuria ≥1.0 g.

During a follow-up period, roughly 7 years after the initial examination, the CP status was re-evaluated in all patients with functioning graft (*n* = 62). The initial and follow-up CPITN values are shown in Table 2. In the follow-up examination, 23 out of 43 (53.5%) patients with mild or no initial periodontitis were assigned to the CPITN 3–4 group. On the other hand, 5 out of 19 (26.3%) patients with initially severe CP were assigned to the CPITN 0–2 group.

#### Univariate Analysis

The results of univariate analysis are given in Table 3.

### 3.2. CP Status and Patient Survival

**Figure 1:**
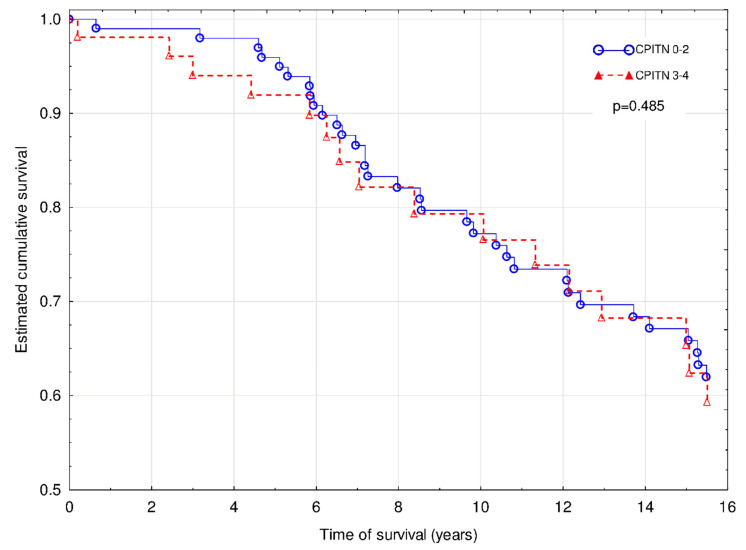
Overall mortality of patients with CPITN (0–2) and CPITN (3–4).

The Cox regression with Firth’s penalized maximum likelihood analysis revealed that age (χ^2^ = 5.3; hazard ratio (HR) 1.04 (95% confidence interval (95% CI) 1.01–1.07; *p* < 0.05), IMT (χ^2^ = 4.0; HR 1.38 (1.01–1.86); *p* < 0.05) and graft loss (χ^2^ = 4.99; HR 1.94 (1.08–3.60); *p* < 0.05) were independently associated with increased risk of overall death (Figure 1). The similar effect of MACE did not reach the statistical significance (χ^2^ = 3.7; HR 1.81 (0.99–3.25); *p* = 0.065).

**Figure 2:**
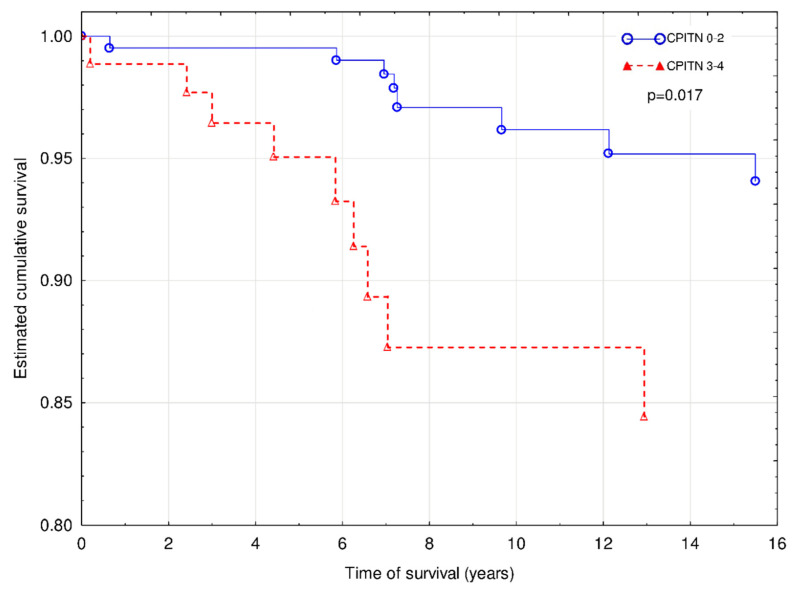
Mortality of patients with a functioning graft with CPITN (0–2) and CPITN (3–4). Covariates: age, sex, IMT, and MACE.

We also investigated the effect of CP status on the death with functioning graft, which was observed in 17 patients (14.5%). The Cox regression with Firth’s penalized maximum likelihood analysis revealed that age (χ^2^ = 4.3; HR 1.07 (1.00–1.15); *p* < 0.05), male sex (χ^2^ = 5.4; HR 5.09 (1.24–46.71); *p* < 0.05), and CPITN (χ^2^ = 5.2; HR 3.54 (1.20–10.45); *p* < 0.05) were independently associated with increased of DWFG (Figure 2). The similar effect of MACE did not reach the statistical significance (χ^2^ = 3.3; HR 2.80 (0.92–8.58); *p* = 0.07).

**Figure 3:**
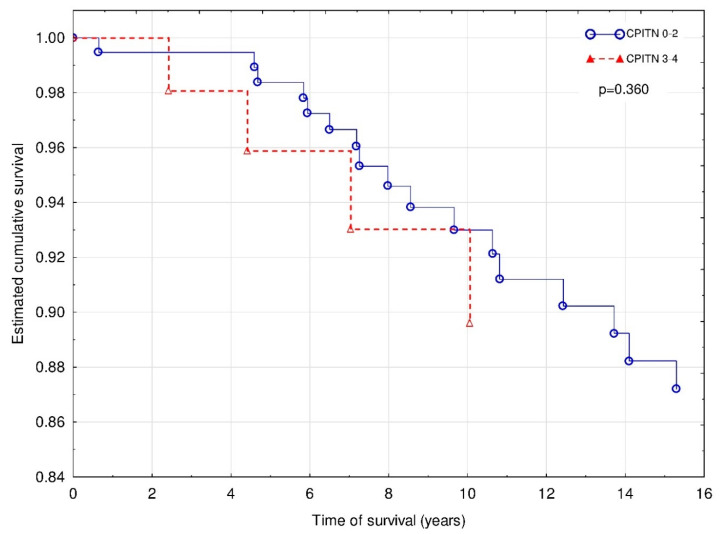
Death caused by CVD events of patients with CPITN (0–2) and CPITN (3–4).

Lastly, the risk of death caused by underlying cardio- or cerebrovascular disease (*n* = 20) was calculated. The Cox regression with Firth’s penalized maximum likelihood analysis revealed that only MACE (χ^2^ = 23.6; HR 9.49 (3.63–30.72); *p* < 0.001) was independently associated with increased risk of CVD death, whereas the effect of age did not reach statistical significance (χ^2^ = 3.8; HR 1.02 (1.00–1.09); *p* = 0.05) (Figure 3).

### 3.3. CP Status and the Risk of MACE

**Figure 4:**
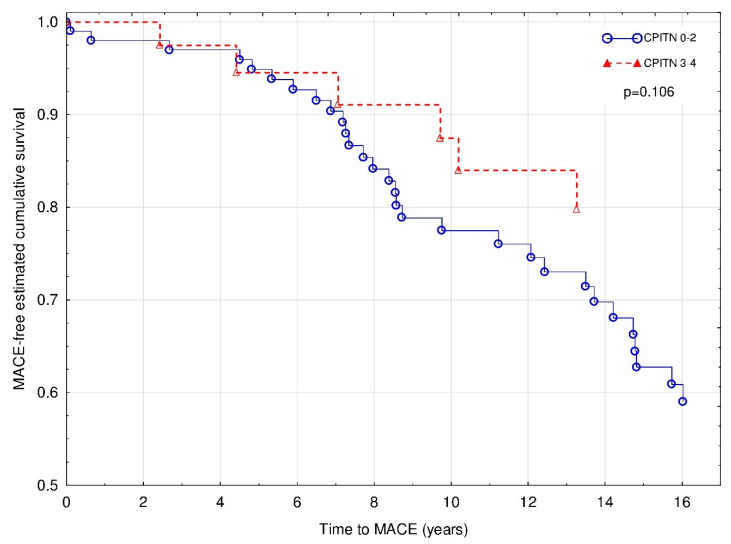
Major cardiovascular events of patients with CPITN (0–2) and CPITN (3–4). Covariates: age, BMI, and HLA class I mismatch.

During the 15-year follow-up period, 60 MACE episodes occurred in 36 patients (30.8%), including fatal complications. Overall, there were 16 strokes, 12 myocardial infarctions, 4 CABG procedures, and 28 single or multiple stent placements. In 19 patients (53%), the first MACE episode was diagnosed with a functioning graft, whereas in 17 patients (47%)—during the dialysis therapy after the kidney graft loss. Out of all MACE episodes, 50% occurred in patients with a functioning graft, and 50% during dialysis therapy. The Cox regression with Firth’s penalized maximum likelihood analysis revealed that age (χ^2^ = 8.3; HR 1.05 (1.01–1.08); *p* < 0.01) and HLA class I mismatch (χ^2^ = 6.2; HR 1.65 (1.11–2.40); *p* < 0.05) were independently associated with increased risk of MACE during the follow-up period (Figure 4). Notably, the negative association between BMI (χ^2^ = 3.8; HR 0.91 (0.83–1.00); *p* = 0.053) and CPITN status (χ^2^ = 2.6; HR 0.52 (0.20–1.14); *p* = 0.11) with MACE occurrence did not reach statistical significance. 

### 3.4. CP Status and the Kidney Graft Loss

**Figure 5:**
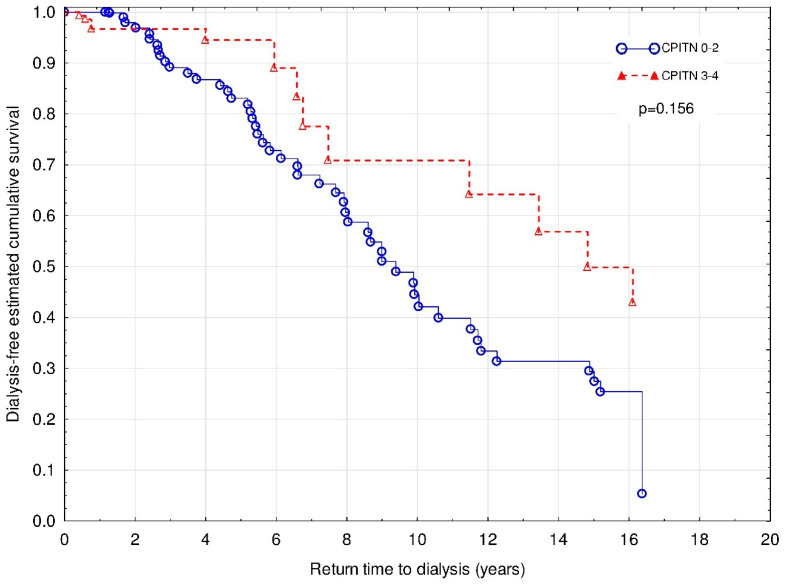
Time to return to hemodialysis for patients with CPITN (0–2) and CPITN (3–4). Covariates: initial serum creatinine, HLA class II mismatch, smoking status, and diastolic BP.

The Cox regression with Firth’s penalized maximum likelihood analysis revealed that serum creatinine concentration at the time of entering the study (χ^2^ = 49.3; HR 1.01 (1.01–1.01); *p* < 0.001), HLA class II mismatch (χ^2^ = 3.9; HR 1.65 (1.00–2.75); *p* < 0.05) and smoking (χ^2^ = 8.3; HR 2.56 (1.37–4.54); *p* < 0.01), but not CPITN status (Figure 5) were independently associated with increased risk of graft loss.

## 4. Discussion

In the present study, we re-evaluated the effect of advanced CP on the 15-year kidney graft and recipient survival in our previously analyzed cohort of kidney transplant patients. After such a long-term follow-up period, we did not find significant influence of the initial periodontal status on all-cause and CVD mortality or kidney graft survival. However, worse periodontal status was independently associated with increased risk of death with a functioning graft. As expected, age and male sex were found to be important confounding factors in the latter analysis.

To our best knowledge, aside from the present study, there is only one study to date which analyzed the relationship between a cross-sectionally assessed oral health and patient and graft survival in the setting of kidney transplantation. Boratyńska et al. reported a numerically higher rate of kidney graft loss and patient mortality in a 10-year follow-up in transplant recipients with gingival overgrowth [13]. However, the methodology was scarcely described, and no survival analyses were performed. Also, cyclosporine-induced gingival overgrowth is not identical to chronic periodontitis. Its pathogenesis is multifactorial and even if periodontal bacteria are involved, gingival hyperplasia could be a symptom of acute rather than chronic periodontitis. Therefore, the results of that study and ours are not necessarily comparable.

In contrast to our recent findings, in the previous analysis of the same cohort, advanced CP was associated with increased risk of death in a 5-year follow-up period [10]. Such a discrepancy may have resulted from the fact that in the present survival analysis we included all patient deaths, also those noted during the dialysis therapy after the kidney graft loss, whereas the return to dialysis program ceased any further observation in the first analysis. The burden of cardiovascular and other risks caused by many factors accumulating during the years, especially uremia-related factors after the loss of the kidney graft function, may mask the negative influence of severe periodontitis on mortality. This is in line with the recent observation that advanced CP increased the risk of DWFG, but not overall mortality. 

The independent influence of periodontal disease on all-cause and cardiovascular mortality [14,15] as well as cardiovascular events [16] was demonstrated in the general population. The chronic inflammatory process, commonly present in individuals with advanced CP, is known to be one of the cardiovascular risk factors [17]. As periodontal bacteria were found in atherosclerotic plaques, this may suggest that that bacteria plays the causal role of CP in the development of vascular lesions [18]. Furthermore, CP is associated with increased blood pressure and left ventricular mass [19,20], also in kidney transplant recipients [21]. To date, it is not certain whether proper dental treatment could modify the kidney graft and patient outcomes in transplanted population. Recently, Santos-Paul et al. reported the reduction of cardiovascular events, coronary events and cardiovascular death in dialysis patients after the periodontal treatment [22]. Moreover, the improvement of kidney excretory function and endothelial function, measured by asymmetric dimethylarginine levels, was observed in CKD patients after periodontal treatment [23]. Although no interventional data are available concerning the kidney transplant population, we may uphold our recommendation from the previous publication [10] that routine oral examination and appropriate dental treatment seems to have a role in risk reduction and should be advised as a standard element of long-term care in kidney graft recipients.

In contrast, the current literature concerning this topic in CKD patients presents contradictory results. Many reports, including a large, NHANES III cohort-based studies, confirmed the association between periodontal disease and all-cause [15,24,25,26] and cardiovascular [15,27] mortality in CKD stages 3–5 patients, whereas other studies did not confirm such a relationship [28,29] or were inconclusive [30,31]. There may be several reasons for such inconsistencies including different stages of CKD, age differences, low number of end-points, or failure of inclusion of important confounding variables in some analyses. In our long-term follow-up study of kidney transplant recipients, we did not confirm an independent influence of CP status on cardiovascular mortality. In fact, the incidence of MACE episodes in patients with advanced CP was lower in our group, and a first MACE episode timing was equivalently distributed between patients with a functioning graft and those who returned to dialysis treatment. However, the presence of CP is only one among numerous, more potent cardiovascular risk factors in this particular population, including traditional (age, hypertension, dyslipidemia) and non-traditional (systemic inflammation, accelerated atherosclerosis, adverse effects of immunosuppressive regimen etc.) ones. It is likely that the postulated independent influence of CP on cardiovascular events and mortality after kidney transplantation warranted further investigation in a larger cohort.

As CP was previously shown to accelerate the progression of CKD [32,33] and increase the acute rejection rate in the first post-transplant year [34], the long-term effect on the kidney graft loss might be anticipated. Until now, only one published study described an association between gingival overgrowth and number of graft loss, but its methodological scarcities and poor statistics limit the usefulness of results. We did not find any relationship between CP status and kidney graft loss in our longitudinal observation. The most probable explanation is the fact that CP may, only to a limited extent, contribute to the development of chronic allograft insufficiency.

The main limitation of this study is the low number of participants. As CP status might be considered as relatively low-potent risk factor of cardiovascular complications and mortality, only a larger multicenter study can definitely confirm its independent influence on the analyzed outcome measures. Another limitation is insufficient longitudinal data concerning the periodontal status and treatment, which hence cannot be adequately analyzed. Besides, many limitations are associated with CPITN itself as a screening method because it gives only a rough estimate of the periodontal treatment needs and does not measure important signs of periodontal disease [35]. Nevertheless, our present study is the first one in literature which analyzed the potential long-term influence of chronic periodontitis on patient and allograft outcomes, including cardiovascular complications, in kidney transplant recipients. Moreover, it was taking into account all deaths, including those noted after patient’s return to dialysis therapy. The timing and nature of death causes and MACE episodes were also analyzed.

In conclusion, in our long-term observation we did not find any evidence that advanced chronic periodontitis independently influenced the risk of all-cause and cardiovascular mortality in patients after renal transplantation. However, CP status was associated with the risk of death with functioning graft in our cohort. An interventional prospective study should be carried out as to whether a routine oral examination and appropriate dental treatment may have a role in risk reduction and should be advised as a standard element of long-term care in kidney graft recipients.

## Figures and Tables

**Table 1:** Clinical characteristics of study patients divided into groups based on the initial CPITN value.

	CPITN 0–2 *n* = 82	CPITN 3–4 *n* = 35	*p*
**At the Time of Initial Dental Examination**			
Age (years)	40.9 (38.5–43.4)	45.8 (42.6–49.1)	<0.05
Sex (M/F)	51/31	26/9	0.21
BMI (kg/m^2^)	24.6 (23.7–25.5)	25.8 (24.3–27.3)	0.15
Dialysis vintage (months) *	24.8 (13.7–43.6)	24.2 (13.1–33.3)	0.49 **
Time since transplantation (months) *	46.7 (30.8–69.3)	36.7 (25.5–68.6)	0.26 **
HLA class I mismatch *	2 (2–3)	2 (2–3)	0.25 **
HLA class II mismatch *	1 (0–1)	1 (1–1)	0.39 **
CIT (h)	21.5 (19.5–23.5)	20.8 (19.3–22.2)	0.65
DGF (*n*, %)	40 (48.8)	17 (48.6)	0.98
Active smoker (*n*, %)	13 (15.9)	8 (22.9)	0.37
Hypertension (*n*, %)	77 (93.9)	33 (94.3)	0.94
Diabetes at study (*n*, %)	3 (3.7)	3 (8.6)	0.27
Systolic BP (mm Hg)	140 (130–150)	145 (140–160)	<0.05
Diastolic BP (mm Hg)	90 (80–90)	95 (90–100)	<0.05
LVMI	112 (105–119)	148 (135–161)	<0.001
IMT (mm)	0.59 (0.57–0.61)	0.63 (0.59–0.67)	0.06
MACE at study (*n*, %)	1 (1.2)	2 (5.7)	0.16
S_cr_ at study (mg/dL) *	1.6 (1.3–2.2)	1.5 (1.2–1.8)	0.31 **
Total cholesterol (mmol/L)	6.0 (5.8–6.3)	6.1 (5.6–6.6)	0.80
Triglycerides (mmol/L)	1.8 (1.6–2.0)	2.1 (1.5–2.7)	0.25
CRP (mg/l) *	0.9 (0.3–1.9)	1.8 (0.7–4.4)	<0.01 **
Calcineurin inhibitor (%)	93.9	91.4	0.47
Steroids (%)	85.4	85.7	0.94
**During the Follow-Up Period**			
Diabetes (*n*, %) ^†^	9 (11.0)	7 (20.0)	0.20
MACE (*n*, %)	30 (36.6)	6 (17.1)	<0.05
Acute rejection (*n*, %) ^†^	30 (38)	8 (22.9)	0.15
CMV infection (*n*, %) ^†^	17 (20.7)	5 (14.3)	0.42
Graft loss (*n*, %)	51 (62.2)	12 (34.3)	<0.01
Cause of graft loss (*n*, %)			
IF/TA	27 (52.9)	7 (58.3)	
GN	12 (23.6)	4 (33.3)	
CR	4 (7.8)	-	
Non-compliance	6 (11.8)	1 (8.4)	0.50
Other	2 (3.9)	-	
DWFG (*n*, %)	8 (9.8)	9 (25.7)	<0.05
Death after graft loss (*n*, %)	25 (30.5)	7 (20.0)	0.25
Cause of death (*n*, %)	33 (40.2)	16 (45.7)	
CVD	16 (48.4)	4 (25.0)	
Infectious	6 (18.2)	6 (37.4)	
Malignancy	6 (18.2)	4 (25.0)	
Accidental	4 (12.2)	1 (6.3)	0.41
Other	1 (3.0)	1 (6.3)	

^†^ All episodes noted since transplantation. Data presented as means and 95% confidence interval or * medians and Q25–Q75 values. Statistics: Student’s *t*-test or χ^2^ test, except ** Mann–Whitney *U* test. CPITN, Community Periodontal Index of Treatment Needs; BMI, body mass index; HLA, human leukocyte antigen; CIT, cold ischemia time; DGF, delayed graft function; BP, blood pressure; LVMI, left ventricular mass index; IMT, intima-media thickness; MACE, major adverse cardiovascular episodes; Scr, serum creatinine concentration; CRP, C-reactive protein; CMV, cytomegalovirus; IF/TA, interstitial fibrosis/tubular atrophy; GN, glomerulonephritis; CR, chronic rejection; DWFG, death with functioning graft; CVD, cardio- or cerebrovascular cause of death.

**Table 2:** Initial and follow-up CPITN values in study patients, in whom the re-evaluation of dental status was performed during the follow-up period.

		CP Group according to the Initial CPITN	
		0–2	3–4
Initial exam (*n* (%))	117 (100)	82 (70.1)	35 (29.9)
Follow-up exam (*n* (%))	62 (53)	43 (69.4)	19 (30.6)
Follow-up CPITN class	[*n* (%)]		
0–2	25 (40.3)	20	5
3–4	37 (59.7)	23	14

CP, chronic periodontitis; CPITN, Community Periodontal Index of Treatment Needs.

**Table 3:** Results of univariate analyses.

	Overall Mortality		DWFG		CVD Mortality		MACE		Graft Loss	
	HR	*p*	HR	*p*	HR	*p*	HR	*p*	HR	*p*
Age	1.06 (1.03–1.09)	<0.001	1.10 (1.04–1.17)	<0.01	1.06 (1.01–1.10)	<0.05	1.03 (0.99–1.06)	0.11		
Sex			9.14 (1.21–68.94)	<0.01						
Dialysis vintage			1.01 (1.00–1.03)	0.08			1.01 (1.00–1.02)	0.10		
HLA MM I							1.65 (1.10–2.48)	<0.05		
HLA MM II									1.68 (1.02–2.76)	<0.05
DGF	1.87 (1.05–3.33)	<0.05	2.12 (0.79–5.75)	0.14						
Smoking	1.60 (0.82–3.13)	0.17							1.91 (1.06–3.43)	<0.05
CRP			1.04 (1.00–1.09)	0.08			1.04 (0.99–1.09)	0.14		
Scr									1.01 (1.01–1.01)	<0.001
SBP	1.03 (1.01–0.15)	<0.05	1.04 (1.00–1.08)	<0.05					1.01 (1.00–1.03)	0.11
DBP	1.03 (1.00–1.07)	<0.05	1.05 (0.99–1.11)	0.11					1.02 1.00–1.05)	0.09
LVMI	1.01 (1.00–1.02)	0.06	1.01 (1.00–1.03)	<0.05						
IMT	1.66 (1.29–2.14)	<0.001	2.16 (1.48–3.16)	<0.001	1.63 (1.10–2.42)	<0.05	1.57 (1.15–2.14)	<0.01		
MACE	2.02 (1.14–3.56)	<0.05	2.81 (1.08–7.30)	<0.05	11.26 (3.78–33.51)	<0.001				
AR									1.46 0.86–2.48)	0.16
Graft loss	1.64 (0.91–2.95)	0.10								
CPITN			2.85 (1.10–7.39)	<0.05			−0.49 (0.20–1.18)	0.11	−0.56 (0.30–1.05)	0.07

DWFG, death with functioning graft; CVD, cardio- or cerebrovascular; MACE, major adverse cardiovascular events; HR, hazard ratio; HLA MM I, human leukocyte antigen class I mismatch; DGF, delayed graft function; CRP, C-reactive protein; Scr, serum creatinine concentration; SBP, systolic blood pressure; DBP, diastolic blood pressure; LVMI, left ventricular mass index; IMT, intima-media thickness; AR, acute rejection; CPITN, Community Periodontal Index of Therapeutic Needs.
